# Development of target focused library against drug target of *P. falciparum* using SVM and Molecular docking

**DOI:** 10.1186/1758-2946-4-S1-P48

**Published:** 2012-05-01

**Authors:** Sangeetha Subramaniam, Monica Mehrotra, Dinesh Gupta

**Affiliations:** 1 Structural and Computational Biology, International Centre for Genetic Engineering and Biotechnology, Aruna Asaf Ali Marg, New Delhi, 110070, India; 2 Department of Computer Science, Jamia Millia Islamia, New Delhi, 110025, India

## 

*PfHslV*, a homolog of β subunit of 20S proteasome forms the proteolytic core of the *PfHslUV* machinery in *P. falciparum *[1,2]. *PfHslV* has no homolog in the human host and it is a promising drug target essential to the plasmodial metabolism. The use of single proteasome inhibitor targeting these threonine proteases has a potential to be antimalarial drug candidate. One of our recent studies identified several promising inhibitors against 20S β5 subunit of *P. falciparum *[3]*.* The present study adopts a similar knowledge based virtual screening strategy using Support Vector Machines (SVM) and molecular docking to build a focused library of potential *PfHslV* inhibitors. SVM model has been trained using 170 molecular descriptors of 64 inhibitors and 208 putative non-inhibitors. The non-linear classifier based on Radial Basis Function (RBF) kernel yielded classification accuracy of 97%. The SVM model rapidly predicted inhibitors from NCI library and were subsequently docked in to the active site of an optimised three-dimensional model of *PfHslV*. The novel drug-like *PfHslV* inhibitors with very good binding affinity and novel scaffold can be a good starting point to develop new antimalarial drugs.
